# Genetic study of quantitative traits supports the use of Guzerá as dual-purpose cattle

**DOI:** 10.5713/ab.21.0458

**Published:** 2022-01-05

**Authors:** Eula Regina Carrara, Maria Gabriela Campolina Diniz Peixoto, Renata Veroneze, Fabyano Fonseca e Silva, Pedro Vital Brasil Ramos, Frank Angelo Tomita Bruneli, Lenira El Faro Zadra, Henrique Torres Ventura, Luiz Antônio Josahkian, Paulo Sávio Lopes

**Affiliations:** 1 Department of Animal Science, Federal University of Viçosa, Viçosa, MG, 36570-900, Brazil; 2 Embrapa Dairy Cattle, Juiz de Fora, MG, 36038-330, Brazil; 3 Brazilian Center for the Genetic Improvement of Guzerá, Belo Horizonte, MG, 30315-380, Brazil; 4 Brazilian Association of Zebu Breeders, Uberaba, MG, 38022-330, Brazil

**Keywords:** Beef Cattle, *Bos indicus*, Dairy Cattle, Genetic Parameters, Sexual Precocity, Guzerá

## Abstract

**Objective:**

The aim of this study was to estimate genetic parameters for 305-day cumulative milk yield and components, growth, and reproductive traits in Guzerá cattle.

**Methods:**

The evaluated traits were 305-day first-lactation cumulative yields (kg) of milk (MY305), fat (FY305), protein (PY305), lactose (LY305), and total solids (SY305); age at first calving (AFC) in days; adjusted scrotal perimeter (cm) at the ages of 365 (SP365) and 450 (SP450) days; and adjusted body weight (kg) at the ages of 210 (W210), 365 (W365), and 450 (W450) days. The (co)variance components were estimated using the restricted maximum likelihood method for single-trait, bi-trait and tri-trait analyses. Contemporary groups and additive genetic effects were included in the general mixed model. Maternal genetic and permanent environmental effects were also included for W210.

**Results:**

The direct heritability estimates ranged from 0.16 (W210) to 0.32 (MY305). The maternal heritability estimate for W210 was 0.03. Genetic correlation estimates among milk production traits and growth traits ranged from 0.92 to 0.99 and from 0.92 to 0.99, respectively. For milk production and growth traits, the genetic correlations ranged from 0.33 to 0.56. The genetic correlations among AFC and all other traits were negative (−0.43 to −0.27). Scrotal perimeter traits and body weights showed genetic correlations ranging from 0.41 to 0.46, and scrotal perimeter and milk production traits showed genetic correlations ranging from 0.11 to 0.30. The phenotypic correlations were similar in direction (same sign) and lower than the corresponding genetic correlations.

**Conclusion:**

These results suggest the viability and potential of joint selection for dairy and beef traits in Guzerá cattle, taking into account reproductive traits.

## INTRODUCTION

The Guzerá cattle breed is one of the most productive among the Zebu breeds present in Brazil and is widely used in crossbreeding, with the aim of producing animals adapted to tropical climatic conditions. In the last 30 years, in addition to genetic selection for beef production, some breeders have included selection for milk yield in Guzerá cattle to obtain economic benefits from the sale of both meat and milk in dual-purpose production systems [1,2].

Dual-purpose cattle systems allow a wide variety of production models because they depend on the preferences of the farmer, local market, household consumption, and local environment, resulting in different proportions of incomes generated from sales of meat and milk [3,4]. Furthermore, systems including both beef and milk production constitute a subsistence system and are an important activity for the economic development of small holders in Latin America [5].

Generally, selection in cattle breeds has allowed large genetic gain that has resulted in beef animals with higher growth rates and dairy cows with higher milk production capacity. In dual-purpose systems, it is important to understand the relationship between growth and milk production traits to evaluate possible genetic antagonisms before defining selection objectives.

Furthermore, genetic progress focused on traits of the highest economic value in cattle was accompanied by declines in animal fertility, making low fertility the main reason for cow disposal [6]. Therefore, it is essential to include reproductive traits in genetic selection processes. In the near future, traits related to feed supply and quality, adaptive robustness, animal welfare, and functionality should also be considered for sustainable selection.

In Brazilian beef cattle, the most commonly used trait as an indicator of fertility and sexual precocity is the scrotal perimeter (SP), which is easy to measure and exhibits a positive genetic correlation with testicular biometry traits (e.g., length, width, and testicular volume), sperm motility and mass activity and a negative genetic correlation with major and total sperm defects [7]. Additionally, age at first calving (AFC) is highly correlated with fertility and reproductive efficiency in both beef and dairy cattle, thus affecting heifer precocity [8,9]. The AFC exhibits heritability of moderate magnitude, approximately 0.20, and favorable (i.e., negative) genetic correlation with milk yield and growth traits in Guzerá cattle [10].

Although the genetic improvement programs of the Guzerá breed for milk and beef are developed independently, joint genetic evaluation is feasible due to the high genetic association between pedigree datasets of the breeding programs [2]. In addition, favorable genetic correlations among dairy (milk yield), growth (weaning weight, yearling weight, and weight at 550 days), and reproductive (AFC) traits were previously reported in Guzerá cattle [10].

Studies on the dual purposes of Guzerá cattle have only recently been conducted. It is expected that joint genetic evaluations considering milk and beef traits will contribute to the genetic improvement of the dual-purpose in this breed. In this context, this study aimed to estimate genetic parameters for 305-day cumulative milk yield and milk components, growth, and reproductive traits in Guzerá cattle.

## MATERIALS AND METHODS

Approval of the animal care and use committee was not needed because this study used existing datasets historically collected by the animal breeding program.

### Animals and data

The phenotypic and pedigree data used in this study were obtained from the Zebu Breeds Genealogical Registry Service (SRGRZ), databases of the Brazilian Association of Zebu Cattle (ABCZ), and the National Program for the Improvement of Guzerá for Dairy Purpose (PNMGuL), jointly coordinated by Embrapa Dairy Cattle and the Brazilian Center for the Genetic Improvement of Guzerá (CBMG^2^).

Records from 197,283 Guzerá males and females born between 1954 and 2018 were used in this study. The pedigree file comprised 545,310 animals, including 148,231 dams and 8,859 sires from 18 generations. The first generation was assumed to be the base population.

The traits evaluated were 305-day first-lactation cumulative yields (kg) of milk (MY305), fat (FY305), protein (PY305), lactose (LY305), and total solids (SY305); AFC in days; adjusted SP (cm) at the ages of 365 (SP365) and 450 (SP450) days; and adjusted body weight (kg) at the ages of 210 (W210), 365 (W365), and 450 (W450) days.

The body weights were adjusted to account for the age of the animal at measurement, age of the dam at birth or weaning, and previous weight, according to the Beef Improvement Federation (BIF) Guidelines [11]. The SP was adjusted to 365 and 450 days by nonlinear logistic regression, a method used in the official genetic evaluations of the breed. A total of 64,050 SP measurements were available from 29,604 animals (one to ten measurements per animal).

To estimate the nonlinear model parameters, the Gauss-Newton iterative method for nonlinear regression (NLIN) in SAS Software [12] was used. The logistic model used is described as follows:


$${SP}_{t}=\frac{A}{1+B\;(\text{exp}(-kt))}+e,$$

where *SP_t_* is the SP at *t* days of age; *A* is the estimated *SP* at maturity; *B* indicates the proportion of mature testis with an asymptotic size to be obtained after birth (established by the initial values of *SP* and *t*); *k* is the maturation index, establishing the rate with which *SP* approaches *A*; and *e* is the random error associated with each measurement. The final estimates of the parameters *A*, *B*, and *k* were 40.1638, 2.7315, and 0.0035, respectively.

Contemporary groups (CGs) for MY305, FY305, PY305, LY305, and SY305 were formed by fitting herd, year, and season of calving. For AFC, SP365, SP450, W210, W365, and W450, the CGs were a combination of herd, year, and season of birth. Additionally, for growth traits, sex was also included in the CG. Both calving and birth seasons were defined as dry (April to September) or rainy (October to March). Data that belonged to the CG with fewer than three records were excluded. The number of CGs per trait and the number of animals per CG are shown in Table 1, which also provides descriptive statistics for each studied trait.

### Genetic analyses

The (co)variance components were obtained by the restricted maximum likelihood method (REML) using AIREMLF90 software [13]. The following general model was assumed:


$$y=\mathrm{Xb}+Z_{1}a+Z_{2}m+Z_{3}\mathrm{mp}+e,$$

where **y** is the vector of phenotypes; **b** is the vector of fixed effects of a CG and covariate; **a** is the vector of random additive direct genetic effects; **m** is the vector of random maternal genetic effects; **mp** is the vector of random maternal permanent environmental effects; **Z_1_**, **Z_2_**, and **Z_3_** are incidence matrices related to the **a**, **m** and **mp** to **y**, respectively; and **e** is the residual vector. The following (co)variance structures were also assumed:


$$\text{Var }\left[\begin{array}{c} a\\ m\\ \mathrm{mp}\\ e \end{array}\right]=\left[\begin{array}{cccc} G_{0\text{a}}⊗A & 0 & 0 & 0\\ v & G_{0\text{m}}⊗A & 0 & 0\\ 0 & 0 & MP_{0}⊗I_{\mathrm{mp}} & 0\\ 0 & 0 & 0 & R_{0}⊗I \end{array}\right],$$

in which **G_0a_** is the additive direct genetic (co)variance matrix; **G_0m_** is the maternal genetic (co)variance matrix; **A** is the numerator relationship matrix; **MP_0_** is the maternal permanent environment (co)variance matrix; **I_mp_** is an identity matrix of order equal to the number of dams with progeny; **R_0_** is the residual (co)variance matrix; **I** is an identity matrix of order equal to the number of animals; and ⊗ is the Kronecker product.

Single-trait (for direct and maternal heritabilities) and bi-trait (for genetic, phenotypic, and residual correlations) analyses were carried out. Specifically, for the correlations between milk production traits (i.e., MY305, FY305, PY305, LY305, and SY305) and SP at different ages (i.e., SP365 and SP450), tri-trait analyses were performed using W210 as an “anchor” trait. This procedure was performed due to the difference in the number of animals phenotyped in these two groups of traits. Therefore, W210 was used to promote better connections between measured traits since it included a greater number of observations and both sexes. The genetic covariances and correlations presented for W210 were derived from bi-trait analyses.

The maternal genetic and permanent environmental effects were adjusted only for W210. The direct maternal genetic covariances were assumed to be zero. The age at calving was considered a linear covariate for MY305, FY305, PY305, LY305, and SY305, and the age of dams at calving was considered a linear covariate for W210, W365, W450, SP365, and SP450.

## RESULTS

The variance components obtained by single-trait analysis for the evaluated traits are provided in Table 2. Direct heritability and genetic and phenotypic correlation estimates among the studied traits are presented in Table 3.

The direct heritability estimates ranged from 0.16 (W210) to 0.32 (MY305). The maternal heritability estimate for W210 was 0.03 (0.004). The genetic correlations among all traits evaluated were favorable. Genetic correlations among milk production traits (milk yield and milk components) were positive and high, ranging from 0.92 (FY305×LY305) to 0.99 (MY305×LY305). For W210, W365, and W450, the genetic correlations were also positive and high, ranging from 0.92 (W210×W365 and W210×W450) to 0.99 (W365×W450). When comparing milk production traits and growth traits, the estimated genetic correlations were moderate, ranging from 0.33 (SY305×W210) to 0.56 (FY305×W365, and PY305 ×W365).

Regarding reproductive traits, the genetic correlations among AFC and all other traits evaluated were negative, presenting values ranging from −0.31 (AFC×FY305) to −0.27 (AFC×SY305) for milk production traits and from −0.43 (AFC×W450) to −0.39 (AFC×W210) for growth traits. Body weights and SP measured at different ages showed positive genetic correlations, ranging from 0.41 (W210×SP450) to 0.46 (W450×SP365). The genetic correlations between SP365 and AFC and between SP450 and AFC were −0.36 and −0.40, respectively. Low-to-moderate genetic correlations were estimated between SP at different ages and milk production traits, ranging from 0.11 (PY305×SP450) to 0.30 (MY305×SP365).

Phenotypic correlation estimates among all traits were similar in direction and smaller than their corresponding genetic correlations. Overall, the phenotypic and residual correlations were similar in direction and magnitude, except between milk production and growth traits. Among milk production traits, the phenotypic correlations were high and positive, ranging from 0.86 (MY305×PY305) to 0.98 (PY305× LY305). All traits presented negative and low-to-moderate phenotypic correlation estimates with AFC. For correlations with milk production traits, values ranged from −0.17 (AFC× SY305) to −0.07 (AFC×MY305), and values ranged from −0.36 (AFC×W365) to −0.27 (AFC×W210) for correlations with growth traits. Phenotypic correlations of AFC with SP365 and SP450 were equal to −0.07 and −0.08, respectively.

Considering growth traits and SP measured at different ages, the phenotypic correlation estimates were positive, presenting moderate magnitudes with values from 0.39 (W210× SP450) to 0.55 (W365×SP365). Growth and SP traits presented positive and low-to-moderate phenotypic correlation estimates with milk production traits, ranging from 0.01 (FY305×W210) to 0.18 (SY305×W365, and SY305×W450). The phenotypic correlation between SP365 and SP450 was positive and high (0.88).

The residual correlation estimates are shown in Table 4. They were high among milk production traits and among growth traits, ranging from 0.84 (MY305×FY305) to 0.98 (PY305× LY305) and from 0.66 (W210×W450) to 0.98 (W365×W450), respectively. Between growth traits and SP traits, the residual correlation estimates were moderate and ranged from 0.42 (W210×SP365, and W210×SP450) to 0.59 (W365×SP365). AFC had a negative residual correlation with all traits evaluated, with values ranging from −0.34 (AFC× W365) to −0.001 (AFC×MY305). The residual correlation estimates between milk production traits and growth traits were low, ranging from −0.15 (PY305×W365) to 0.05 (SY305× W365). When different traits are not observed in the same individual, there is no residual covariance between the traits [14]. Thus, residual correlation estimates between milk production traits and SP traits and between AFC and SP traits were not determined.

## DISCUSSION

This study estimated genetic associations among milk yield, milk components, growth traits, and reproductive traits in Guzerá cattle. To the best of our knowledge, the study by Brito et al [10] is the only study reporting the genetic correlations among milk, growth, and reproductive traits in this breed. In the present study, however, a larger number of animals and important traits, such as milk components and SP, for the genetic improvement of dual-purpose Guzerá were included in the analyses. Milk components, especially major components, i.e., fat, protein, and lactose, are important because they directly affect milk properties in industrial processing and in the quality of dairy products. In addition, SP is important because it is easy to measure and has a negative (favorable) genetic correlation with sexual precocity in both males and females [15,16], rendering it a potential selection criterion for herds with sexual precocity. Thus, the present study is conclusive and of great importance for the genetic improvement of the breed.

Overall, heritabilities were moderate (0.16 to 0.32), indicating that all traits studied could respond to selection with lesser or greater intensity and could achieve a satisfactory genetic progress rate in the breeding program of Guzerá cattle. The heritability estimate for MY305 (0.32) was similar to that reported by Brito et al [10] (0.29), and it was higher than those reported by Santos et al [17] (0.24) and Gama et al [18] (0.24) in studies also conducted on Guzerá cattle. It must be highlighted that heritability is a property of a population and its mating system in a specific environment and time. Different heritability estimates among studies on the same breed can be explained by differences in the sample size, type of records used in each study (e.g., first or multiple lactations), and estimation methods. Heritability estimates for milk components (0.22 to 0.31) were greater than those obtained by Silva et al [19] in Guzerá cattle. Although reproductive traits are strongly influenced by environmental components, the heritability estimate for AFC was moderate (0.20). Our results reflect, however, that this trait in Guzerá cattle has a larger genetic effect; thus, selection for improvements in reproductive performance is possible. The breeding value of AFC was recently included in the Guzerá sire summary to increase precocity; therefore, we expect to soon be able to evaluate the genetic response to selection. This is an important trait to be included as a selection goal since the Guzerá breed is still reproductively late. Regarding SP365 and SP450, the heritability estimates were moderate (0.20 and 0.21, respectively) and were lower than those found in the literature for beef cattle, including Guzerá [16,20].

Regarding growth traits, the heritability estimates were moderate (0.16 to 0.24). They corroborated the estimates reported by Gama et al [18], but they were lower than those in most of the consulted literature for Guzerá cattle [10,20]. Although low, the maternal heritability for W210 (0.03) was similar to that reported by other studies on Guzerá cattle [10,20].

Animal breeding strategies are determined by the relative importance of traits and the genetic correlation between traits. With regard to the dual-purpose focus, there have been few studies that have addressed genetic relationships between dairy and beef traits. The genetic correlations between MY305 and 305-day cumulative milk components were high (0.92 to 0.99). No studies have reported genetic correlations between MY305 and 305-day cumulative milk components in Guzerá cattle. A high genetic correlation indicates a strong genetic association among these traits, i.e., selection for higher MY305 promotes increased 305-day cumulative milk components.

Among growth traits, the genetic correlations were high (0.92 to 0.99), emphasizing that most genes controlling weight at a given age are the same at other ages, making it possible to select animals at younger ages. The genetic correlations among growth traits found in the present study corroborate the literature on cattle breeds [10,20,21].

The genetic correlation estimates between AFC and all evaluated traits were low to moderate and favorable. Between AFC and MY305, the genetic correlation was equal to −0.28. Although of moderate magnitude, this result suggests that females selected for MY305 would exhibit improved sexual precocity. To the best of our knowledge, no other studies on other Zebu cattle breeds have reported genetic correlations between AFC and MY305. Genetic selection involving milk yield and AFC should be performed with caution to avoid an increasing risk of dystocia in younger cows, mainly those with high genetic advantage for milk yield [22]. The genetic correlations among AFC and milk components were low (−0.31 to −0.27) but favorable, suggesting that the selection process for AFC would result in a milk yield with higher 305-day cumulative solid content or vice versa. Thus, considering the high estimates obtained for heritabilities of milk constituents in this study, the selection for these traits could yield higher genetic gains.

Genetic correlations between AFC and growth traits were low but favorable (−0.43 and −0.39). In turn, genetic correlations between AFC and SP at different ages (i.e., SP365 and SP450) were −0.36 and −0.40, respectively, which are values consistent with those reported in the literature for beef cattle [16,21]. The endocrine axis regulating puberty in bulls and heifers is similar, which may explain the existence of genetic correlations between male and female reproductive traits, which can be exploited in breeding programs [23,24]. Thus, considering all traits of interest for the improvement of Guzerá cattle, it is possible to reach the highest genetic progress for female precocity using SP as a selection criterion without impairing genetic progress regarding body weight.

Regarding SP, the genetic correlations with MY305 and 305-day cumulative milk components (0.11 to 0.30) were low to moderate, suggesting little genetic association among these traits. Thus, selection for MY305 and milk components will not increase SP but will not cause harm either. Some studies reported genetic correlations among SP and growth traits, with estimates ranging from 0.30 to 0.78 [20,21]. In the present study, the genetic correlations between SP traits and growth traits were moderate and ranged from 0.41 to 0.46, indicating that selection to increase body weights at different ages also increases SP365 and SP450 and vice versa. The positive and favorable genetic correlation between SP365 and SP450 found in the present study (0.96) and in other studies on beef cattle (0.90 to 0.94) [16,20] indicated that one-year-old bulls with a higher SP also had a higher SP at a yearling age. The genetic correlations involving SP365 suggest that its use as a selection criterion in breeding programs is advantageous because, in addition to allowing the early selection of animals, it does not promote losses to the other traits, with the possibility of a genetic response associated with female precocity.

The genetic correlations between milk production and growth traits were moderate and favorable (0.33 to 0.56) and were highest among W365 and milk production traits (i.e., MY305, FY305, PY305, LY305, and SY305), ranging from 0.44 to 0.56. These results suggest that selection for MY305 and milk components would also result in animals with a higher weight at one year of age. Few studies have evaluated genetic relationships between milk and growth traits in cattle, but our results suggest the existence of pleiotropic effects among these traits. The estimates found in the current study are slightly higher than those reported by Gama et al [18] and Brito et al [10], both with Guzerá cattle.

Specifically, for milk components genetically correlated with AFC, growth traits, and SP traits, the standard errors of the estimates were high. This can be explained by data-related differences among the traits, with milk components having a much lower number of observations and fewer animals with phenotypes for all traits evaluated. The standard errors were slightly higher for genetic correlations between milk components and SP traits, possibly due to the difference in the number of animals recorded for the two groups of traits. Thus, the genetic correlation estimates among these traits should be interpreted with caution.

The phenotypic correlation estimates exhibited a similar trend to the corresponding genetic correlation estimates; however, they generally had lower magnitudes than the genetic correlations. Phenotypic correlation estimates among milk, growth, and reproductive traits were smaller in magnitude than genetic correlation estimates in other studies involving genetic parameter estimates in dual-purpose breeds [10,25]. A phenotypic correlation lower than its corresponding genetic correlation with a small positive residual correlation can be explained by the fact that the genes underlying the two traits are similar, but the environments in which they express and that influence these traits have a low correlation [26]. In the current study, this condition occurred only among SY305 and growth traits. Comparing the milk production traits (i.e., MY305, FY305, PY305, LY305, and SY305), the residual correlations were positive and high (0.84 to 0.98), suggesting that residual effects (i.e., nonadditive and environmental) similarly influence these traits. For these traits, the genetic and their corresponding phenotypic correlations were similar in sign and magnitude. The same trend occurred among the beef traits (i.e., W210, W365, W450, SP365, and SP450), where the residual correlations ranged from 0.42 to 0.98.

The confidence of breeders in the genetic evaluation results of the National Program for the Improvement of Guzerá for Dairy Purpose has increased the number of herds and animals participating in this program, as well as the use of this breed in dual-purpose production systems [27]. The nonantagonism among milk, growth and reproductive traits in Guzerá cattle makes simultaneous selection for beef and dairy traits feasible. Each breeder has the option to specialize the herd for milk, beef, or both purposes. The genetic gains associated with each trait will not be the same as those obtained by direct selection for only one of them, but joint selection is possible.

## CONCLUSION

All evaluated traits presented moderate heritability estimates and the possibility of responding to selection in the Guzerá breed.

Furthermore, the favorable genetic correlations among all evaluated traits suggest the possibility of joint genetic selection for milk and beef production in Guzerá cattle, with a favorable correlated response for reproductive traits.

## Figures and Tables

**Table 1:** Number of observations (N), mean, standard deviation (SD), coefficient of variation (CV, %), minimum (MIN), maximum (MAX), number of contemporary groups (CGs), and range of number of animals by CG (N by CG) included in the analyses of each trait

Traits^1)^	N	Mean	SD	CV	MIN	MAX	CG	N by CG
MY305 (kg)	5,229	1,991.17	980.90	49.26	105.00	6,487.00	516	3 to 67
FY305 (kg)	1,853	82.82	37.06	44.74	6.00	281.17	181	3 to 51
PY305 (kg)	1,543	62.08	27.16	43.74	4.00	232.00	113	3 to 51
LY305 (kg)	1,457	77.50	34.15	44.07	5.00	226.00	108	3 to 51
SY305 (kg)	1,228	227.02	96.30	42.42	13.00	722.00	93	3 to 51
AFC (d)	83,244	1,251.50	208.84	16.69	671.00	1,680.00	9,592	3 to 197
W210 (kg)	122,684	173.70	39.92	22.98	50.00	300.00	9,974	3 to 188
W365 (kg)	88,065	227.41	53.36	23.46	67.00	414.00	7,683	3 to 169
W450 (kg)	88,456	275.75	58.58	21.25	102.27	499.00	7,710	3 to 169
SP365 (cm)	26,988	21.55	3.45	15.99	9.17	33.98	1,511	3 to 229
SP450 (cm)	27,047	23.85	3.75	15.74	10.25	37.98	1,519	3 to 230

1) MY305, 305-day milk yield; FY305, 305-day fat yield; PY305, 305-day protein yield; LY305, 305-day lactose yield; SY305, 305-day total solid yield; AFC, age at first calving; W210, 210-day weight; W365, 365-day weight; W450, 450-day weight; SP365, 365-day scrotal perimeter; SP450, 450-day scrotal perimeter.

**Table 2:** Estimates of variance components and their standard errors (within parentheses) for 305-day milk yield, 305-day milk components, growth traits, and reproductive traits studied in Guzerá cattle obtained by single-trait analysis.

Traits^1)^	$\sigma_{a}^{2}$	$\sigma_{m}^{2}$	$\sigma_{mp}^{2}$	$\sigma_{e}^{2}$
MY305	158,150.00 (20,124.00)	-	-	340,320.00 (15,534.00)
FY305	202.53 (51.61)	-	-	714.74 (45.89)
PY305	144.11 (33.11)	-	-	345.67 (26.90)
LY305	251.66 (57.49)	-	-	570.79 (46.45)
SY305	2,037.10 (513.24)	-	-	4,621.40 (421.14)
AFC	5,912.50 (264.82)	-	-	23,596.00 (222.58)
W210	109.05 (5.67)	20.64 (2.64)	70.09 (3.08)	493.48 (4.18)
W365	304.12 (12.46)	-	-	952.60 (9.68)
W450	360.46 (14.98)	-	-	1,194.40 (11.71)
SP365	1.48 (0.13)	-	-	5.83 (0.10)
SP450	1.78 (0.15)	-	-	6.89 (0.12)

1) $\sigma_{a}^{2}$, additive genetic variance; $\sigma_{m}^{2}$, maternal genetic variance; $\sigma_{mp}^{2}$, maternal permanent environmental variance; $\sigma_{e}^{2}$, residual variance; MY305, 305-day milk yield; FY305, 305-day fat yield; PY305, 305-day protein yield; LY305, 305-day lactose yield; SY305, 305-day total solid yield; AFC, age at first calving; W210, 210-day weight; W365, 365-day weight; W450, 450-day weight; SP365, 365-day scrotal perimeter; SP450, 450-day scrotal perimeter.

**Table 3:** Direct heritability estimates (diagonal), genetic correlations (above diagonal) and phenotypic correlations (below diagonal), as well as their standard errors (within parentheses), for 305-day milk yield, 305-day milk components, growth traits, and reproductive traits studied in Guzerá cattle obtained by bi-trait and tri-trait analyses^1)^

Traits^2)^	MY305	FY305	PY305	LY305	SY305	AFC	W210	W365	W450	SP365	SP450
MY305	0.32 (0.04)	0.96 (0.024)	0.97 (0.016)	0.99 (0.001)	0.98 (0.013)	−0.28 (0.079)	0.35 (0.081)	0.44 (0.075)	0.40 (0.078)	0.30** (0.100)	0.26** (0.102)
FY305	0.86 (0.006)	0.22 (0.05)	0.95 (0.025)	0.92 (0.034)	0.98 (0.014)	−0.31 (0.139)	0.43 (0.122)	0.56 (0.105)	0.54 (0.107)	0.22** (0.161)	0.16** (0.165)
PY305	0.90 (0.004)	0.91 (0.005)	0.29 (0.06)	0.96 (0.013)	0.98 (0.009)	−0.29 (0.138)	0.42 (0.124)	0.56 (0.103)	0.53 (0.117)	0.22** (0.155)	0.11** (0.123)
LY305	0.92 (0.003)	0.90 (0.006)	0.98 (0.001)	0.31 (0.06)	0.98 (0.009)	−0.29 (0.138)	0.37 (0.129)	0.53 (0.108)	0.50 (0.111)	0.26** (0.156)	0.17** (0.163)
SY305	0.92 (0.004)	0.96 (0.003)	0.96 (0.003)	0.95 (0.003)	0.31 (0.07)	−0.27 (0.151)	0.33 (0.141)	0.50 (0.119)	0.47 (0.122)	0.22** (0.171)	0.13** (0.178)
AFC	−0.07 (0.021)	−0.12 (0.035)	−0.12 (0.039)	−0.11 (0.040)	−0.17 (0.042)	0.20 (0.01)	−0.39 (0.034)	−0.41 (0.030)	−0.43 (0.031)	−0.36** (0.049)	−0.40** (0.047)
W210	0.06 (0.031)	0.01 (0.046)	0.05 (0.050)	0.04 (0.051)	0.09 (0.057)	−0.27 (0.007)	0.16 (0.01)	0.92 (0.007)	0.92 (0.008)	0.45 (0.046)	0.41 (0.046)
W365	0.09 (0.034)	0.07 (0.050)	0.06 (0.055)	0.08 (0.057)	0.18 (0.062)	−0.36 (0.007)	0.72 (0.002)	0.24 (0.01)	0.99 (0.001)	0.44 (0.042)	0.42 (0.042)
W450	0.08 (0.033)	0.07 (0.049)	0.08 (0.054)	0.10 (0.056)	0.18 (0.061)	−0.35 (0.007)	0.71 (0.002)	0.99 (0.001)	0.23 (0.01)	0.46 (0.042)	0.44 (0.042)
SP365	0.08 (0.026)	0.05 (0.037)	0.06 (0.040)	0.07 (0.041)	0.06 (0.044)	−0.07 (0.010)	0.40 (0.007)	0.55 (0.006)	0.54 (0.006)	0.20 (0.02)	0.96 (0.007)
SP450	0.07 (0.027)	0.04 (0.038)	0.03 (0.042)	0.05 (0.043)	0.03 (0.046)	−0.08 (0.010)	0.39 (0.007)	0.54 (0.006)	0.53 (0.006)	0.88 (0.002)	0.21 (0.02)

1) Exclusively for the correlation between milk production traits (i.e., MY305, FY305, PY305, LY305, and SY305) and scrotal perimeter at different ages (i.e., SP365 and SP450); tri-trait analyses were performed using W210 as the “anchor” trait, e.g., for the comparison between MY305 and SP365, MY305×W210×SP365 was performed and analogous for the others. For all other traits, bi-trait analyses were performed in a two-by-two manner. The genetic parameters presented for W210 were obtained from the bi-trait analyses.

2) MY305, 305-day milk yield; FY305, 305-day fat yield; PY305, 305-day protein yield; LY305, 305-day lactose yield; SY305, 305-day total solid yield; AFC, age at first calving; W210, 210-day weight; W365, 365-day weight; W450, 450-day weight; SP365, 365-day scrotal perimeter; SP450, 450-day scrotal perimeter.

**Table 4:** Residual correlations and their standard errors (within parentheses) for 305-day milk yield, 305-day milk components, growth traits, and reproductive traits estimated for Guzerá cattle obtained by bi-trait analyses

Traits^1)^	MY305	FY305	PY305	LY305	SY305	AFC	W210	W365	W450	SP365	SP450
MY305	-	0.84 (0.011)	0.89 (0.008)	0.89 (0.007)	0.90 (0.007)	−0.001 (0.030)	−0.03 (0.047)	−0.05 (0.050)	−0.05 (0.048)	-	-
FY305		-	0.90 (0.009)	0.89 (0.010)	0.93 (0.007)	−0.07 (0.047)	−0.14 (0.067)	−0.11 (0.073)	−0.09 (0.071)	-	-
PY305			-	0.98 (0.002)	0.95 (0.054)	−0.07 (0.051)	−0.06 (0.076)	−0.15 (0.085)	−0.11 (0.083)	-	-
LY305				-	0.94 (0.006)	−0.05 (0.056)	−0.07 (0.079)	−0.12 (0.089)	−0.07 (0.086)	-	-
SY305					-	−0.14 (0.059)	0.02 (0.087)	0.05 (0.096)	0.06 (0.093)	-	-
AFC						-	−0.27 (0.009)	−0.34 (0.010)	−0.33 (0.010)	-	-
W210							-	0.67 (0.003)	0.66 (0.003)	0.42 (0.011)	0.42 (0.011)
W365								-	0.98 (0.0002)	0.59 (0.011)	0.58 (0.011)
W450									-	0.56 (0.010)	0.56 (0.010)
SP365										-	0.86 (0.003)
SP450											-

1) MY305, 305-day milk yield; FY305, 305-day fat yield; PY305, 305-day protein yield; LY305, 305-day lactose yield; SY305, 305-day total solid yield; AFC, age at first calving; W210, 210-day weight; W365, 365-day weight; W450, 450-day weight; SP365, 365-day scrotal perimeter; SP450, 450-day scrotal perimeter.
